# Supplementary material for: Combination of Zearalenone and Deoxynivalenol Induces Apoptosis by Mitochondrial Pathway in Piglet Sertoli Cells: Role of Endoplasmic Reticulum Stress
Source: Toxins (Basel). 2023 Jul 21;15(7):471. doi: 10.3390/toxins15070471 (PMC10467067; doi:10.3390/toxins15070471)
Supplement: Supplementary file 1 [file toxins-15-00471-s001.zip › toxins-2449262-supplementary.pdf]

Table S1. Primers for different genes used in this study.

Genes	Accession Number	Primer	Primer sequences（5'→3'）	Product Size/bp
GAPDH	NM_001206359.1	Forward	TGACCCCTTCATTGACCTCC	160
		Reverse	CCATTTGATGTTGGCGGGAT	
Bid	NM_001030535.1	Forward	CAACGGTGAACTTCATCAAC	70
		Reverse	CAGTCCATCTCACTTTGGAC	
Bcl-2	XM_021099593.1	Forward	TCCAGGCAGTTTAATACATTC	80
		Reverse	TCCCTTTATACACTGGGTGA	
Caspase9	XM_013998997.2	Forward	GAGACAGCTCAAATTGCAGG	101
		Reverse	TTCGCCTCTCTTTCTCCATC	
Mfn2	XM_021095349.1	Forward	CGGGAAGGTGAAGAATTGGA	127
		Reverse	CTTCACAGGGTAGGCATCAT	
Drp1	XM_021092060.1	Forward	TGGCTAATAGAAATGGAACA	120
		Reverse	GATACTGAGCAGCAAGAACA	
P53	NM_213824.3	Forward	GAGCTGCAATGGAGGAGTC	85
		Reverse	AGCAGTTTCCACAAGTCTGA	
